# Halitosis, reduced salivary flow and the quality of life in pre-kidney transplantation patients

**DOI:** 10.4317/jced.57282

**Published:** 2020-11-01

**Authors:** Natalia-Garcia Santaella, Aloizio-Premoli Maciel, Guilherme Simpione, Paulo-Sérgio-da Silva Santos

**Affiliations:** 1 Department of surgery, stomatology, pathology and radiology, Bauru School of Dentistry, University of São Paulo, Bauru, Brazil

## Abstract

**Background:**

Halitosis is a condition that affects 50% of adults and one third of patients with chronic kidney disease. The aim of this study was to evaluate oral manifestations and volatile sulfur compounds (VSCs) associated with halitosis and quality of life in pre-kidney transplantation candidates.

**Material and Methods:**

The organoleptic test (OT) and halimetry (HA) (before and after cysteine mouthwash) were performed in patients with the Oralchroma® device, stimulated (SE) and non-stimulated sialometry (SN), Tongue Coating Index (TCI). The OHIP-14 questionnaire was administered to assess the impact of oral health on quality of life.

**Results:**

Fourteen individuals with a mean age of 49.64 ± 13.35 years were evaluated. In the organoleptic test, 57.14% of the individuals presented halitosis. Oralchroma results showed that dimethyl sulfide was above the threshold in 85.71% of the individuals, while hydrogen sulfide and methyl mercaptan were above threshold in 28.57%; after the use of cysteine, hydrogen sulfide was present in 100% of the cases, dimethyl sulfide in 57.14% and methyl mercaptan in 50%. In the non-stimulated sialometry, 57.14% of the individuals presented hyposalivation and 21.42% in the stimulated. Regarding the tongue coating index, 100% of the individuals presented tongue coating, with a mean of 7.64. The assessment of impact of oral health on quality of life showed a negative impact in all dimensions.

**Conclusions:**

Tongue coating, in association with hydrogen sulfide, was the main cause of halitosis in the study subjects, and hyposalivation may contribute to higher tongue-coating indices. These oral changes negatively affect the quality of life for pre-kidney transplantation patients.

** Key words:**Halitosis, chronic kidney disease, quality of life, kidney transplantation.

## Introduction

Renal replacement therapy is performed after chronic kidney failure in order to reestablish some kidney functions (1). Although renal replacement therapy is performed to treat chronic kidney disease (CKD), the only treatment modality that can reestablish kidney functions (2) and restore the quality of life (QOL) of the patients is kidney transplantation (KT) (3).

Despite all of the benefits provided by KT to the recipients, there are some comorbidities and oral manifestations that occur due to the use of immunosuppressive drugs related to CKD and to the additional medications used, for example, hyposalivation and its consequences, such as dry month, tongue coating and halitosis (4,5). Halitosis is one of the relevant oral manifestations observed in individuals with CKD before and after KT.

There are several methods to measure and to establish a diagnosis of halitosis, but Oralchroma® is the only halimetry test that can identify the volatile sulfur compounds (VSCs) responsible for halitosis (6,7). The identification of VSCs is important to the establishment of the etiology and treatment of halitosis and, consequently (8), improving the QOL of individuals with CKD.

Available articles evaluating halitosis in KT candidates are scarce, especially when it comes to studies using Oralchroma®. Therefore, the aim of this study was to evaluate oral manifestations and VSCs associated with halitosis in KT candidates.

## Material and Methods

A cross-sectional study was performed in patients with chronic kidney disease undergoing hemodialysis in the pre-kidney transplantation phase. This study was approved by the Research Ethics Committee of the institution where it was carried out (CAEE 71651517.9.000.5417).

The inclusion criteria were as follows: age over 18 years and diagnosis of chronic kidney disease undergoing hemodialysis three times a week who agreed to participate in the study and signed the informed consent form. The exclusion criteria were as follows: known history of head and neck cancer and/or radiotherapy in this region, current diagnosis of sinusitis, lower respiratory tract infection, gastric reflux and/or liver failure, use of medications including antihistamines, tricyclic antidepressants and antibiotics four weeks prior to the dental appointment.

Before the evaluation, the research subjects were instructed to go 24 hours without eating spicy foods and/or those with a very strong odor and to not drink alcoholic beverages. Three hours before the consultation, subjects were instructed to avoid brushing their teeth, flossing, chewing gum and smoking. At the dental appointment time, subjects were instructed to not use cosmetics with odors and to not eat or drink for at least one hour before the dental appointment time (9).

The organoleptic test was performed by a previously trained and calibrated examiner as recommended (10) during which the examiner assessed the patient’s breath odor 10 cm away from the patient and determined the rate as follows: 0 = none, 1 = barely noticeable, 2 = mild but clearly noticeable, 3 = moderate, 4 = strong and 5 = extremely strong.

Volatile sulfur compounds were measured using the OralChroma™ gas chromatograph (FIS, Itami, Hyogo, Japan) according to the instruction manual in two separate circumstances. During the first measurement, there was no influence from any external factors at the beginning of the consultation. The second measurement occurred after a cysteine mouthwash (10 ml of 6-mM cysteine, pH 7.2) for 30 seconds, as a counter test to induce the formation of VSCs if patients were colonized with halitosis-related bacteria (11,12). Thresholds of 112 for hydrogen sulfide, 26 for methyl mercaptan and 8 for dimethyl sulfide were considered (13).

Stimulated and unstimulated salivary flow were measured (14), and the amount was classified (15) as normal, hyposalivation and very low hyposalivation.

Tongue coating was measured by using the tongue coating index (16), which divides the tongue into six quadrants and each quadrant receives a score from 0 to 2 (0 being uncoated, 1 lightweight and 2 severe) and totaling a maximum score of 12.

The questionnaire OHIP-14 (Oral Health Impact Profile) analyzing the dimensions of oral health impact on quality of life was administered and consisted of seven dimensions and 14 questions, developed by Slade (17) and validated in Portuguese (18). Quality of life factors included functional limitation, physical pain, psychological discomfort, physical disability, psychological disability, social disability and handicap.

Descriptive statistics was performed.

## Results

27 individuals with chronic kidney disease undergoing hemodialysis were evaluated and of these 14 were in the pre-kidney transplant phase and were included in this study. They were nine men and five women, with a mean age of 49.64 years. The time of hemodialysis of these individuals was an average of 48.5 months.

The organoleptic test showed that 57.14% of individuals had halitosis, characterizing them with a noticeable odor (average 2.5). The OralChroma test revealed dimethyl sulfide in 85.71% of the subjects, while hydrogen sulfide and methyl mercaptan were detected in 28.57%, both tests without the cysteine mouthwash. In the cysteine post-mouthwash measurement, hydrogen sulfide was present in 100% of cases, dimethyl sulfide in 57.14% and methyl mercaptan in 50%.

Unstimulated sialometry showed hyposalivation in 57.14% of individuals, while stimulated sialometry showed hyposalivation in 21.42%. When assessing the tongue coating index, 100% of the individuals presented a mean tongue coating of 7.64.

The mean of the VSC, tongue coating index, organoleptic test, unstimulated sialometry and stimulated sialometry as well as the percentage of subjects with halitosis, tongue coating and unstimulated and stimulated hyposalivation are shown in Table 1.

**Table 1:**
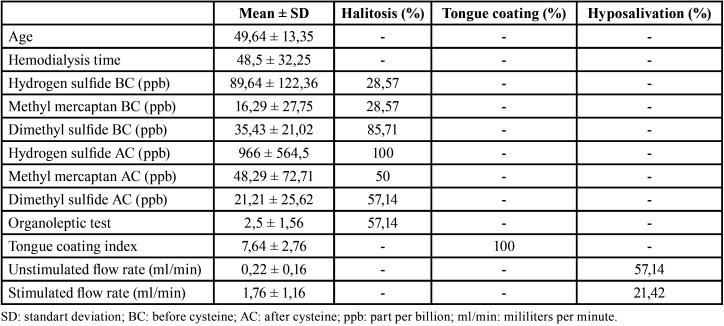
Mean and standart deviation of age, hydrogen sulfide, methyl mercaptan and dimethyl sulfide (before and after cysteine), organoleptic test, tongue coating index, unstimulated and stimulated flow rate and percentage of subjects with halitosis, tongue coating and hyposalivation.

The assessment of impact of oral health on quality of life showed a negative impact on the dimensions of functional limitation, physical disability, social disability and handicap and a mean on the dimensions of physical pain, psychological discomfort and psychological disability (Table 2).

**Table 2:**
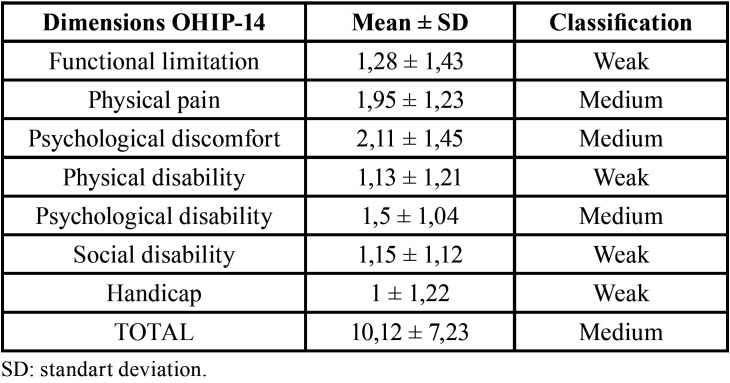
Mean, standart deviation and classification of dimensions of OHIP-14halitosis, tongue coating and hyposalivation.

## Discussion

Halitosis was observed in individuals with chronic kidney disease by both the organoleptic test and gas chromatography. The levels of dimethyl sulfide were high before the cysteine mouthwash, while hydrogen sulfide and methyl mercaptan levels increased and dimethyl sulfide decreased after the cysteine mouthwash, demonstrating that these patients have intraoral halitosis caused by tongue coating. Hydrogen sulfide is the main compound related to tongue coating (8,19), followed by methyl mercaptan (8,20), and tongue coating was also found at high rates in more than half of the patients.

Although dimethyl sulfide is mainly related to systemic halitosis (8), its reduction after the cysteine mouthwash and the associated increase in hydrogen sulfide shows that halitosis in these patients is of intraoral origin. Despite the existence of a relationship between systemic halitosis and chronic kidney disease, this is more commonly observed in cases where urea levels are decompensated (21). Urea levels were stabilized by renal replacement therapy in the subjects of our research project by hemodialysis three times a week. Another contributing factor to these results was the assessment that occurred on days interspersed with hemodialysis, where low levels of uremia are expected.

The presence of VSCs may also be related to a reduced salivary flow that promotes halitosis (22) through increased tongue coating (6), which is directly associated with hydrogen sulfide (19). Such cases were observed in 100% of the individuals in our study after cysteine mouthwash, probably because our study is unprecedented in use of cysteine as halitosis counter test in these patients.

Tongue coating is primarily caused by poor tongue hygiene and may also be associated with reduced salivary flow (6), which was observed in our study that all patients showed tongue coating and more than half patients (57,14%) having presented hyposalivation (unstimulated flow rate). Reduced salivary flow is caused by CKD-associated changes in the salivary glands and reduced fluid intake in these patients (23,24).

Studies have shown that halitosis can have a negative impact on quality of life (25-28), but none of them used the OHIP-14 questionnaire and their respective interpretations as it was used in this research. Consistently, we observed a negative impact of oral health on quality of life in all dimensions of OHIP-14, and this impact may be related to both the halitosis and reduced salivary flow present in most subjects of this study.

The negative impact of halitosis on quality of life may affect the psychological sphere as observed in a previous study (28) and in our study, mainly when applied OHIP-14. The dimensions that were most affected in individuals with halitosis were psychological discomfort, psychological disability and disability. These data are important clinically because a simple orientation of tongue hygiene can improve the quality of life of patients with tongue coating-related halitosis.

The reduction in salivary flow also seems to have impacted the quality of life on the functional limitation dimension due to the damage that the reduction in saliva can cause on chewing, swallowing and speech (23,29) of these individuals. In our study, more than half patients presented a reduction in unstimulated salivary flow, representing an alert for health professionals, that should be aware of the signs of dry mouth, halitosis and the possible complaint of xerostomia for the institution of the appropriate therapy.

Tongue coating associated with the production of hydrogen sulfide is the main cause of halitosis in individuals with chronic kidney disease in the pre-kidney transplant phase. Hyposalivation may be a factor that contributes to higher levels of tongue coating and consequently to the occurrence of halitosis in these individuals. These oral changes may contribute to discomfort and psychological disability and negatively affect the quality of life before kidney transplantation.
